# Swelling of the Muscles of the Shoulder Girdle in a CT Scan is a Leading Finding for the Diagnosis of Polymyositis

**DOI:** 10.5334/jbsr.3296

**Published:** 2023-11-09

**Authors:** Ali Al-Ani, Sadeq Da’meh, Amin Da’meh

**Affiliations:** 1 UZ Brussel, BE; 2 The Jordanian Royal Medical Services, JRMS, Jordan

**Keywords:** myositis, muscular pain, muscular edema, PET-CT, computed tomography

## Abstract

Polymyositis (PM) is an uncommon inflammatory disease of unknown cause, but the disease shares many characteristics with autoimmune disorders. In the past, the diagnosis criteria for PM depended primarily on clinical features, blood enzyme levels, an electromyogram, and muscle biopsies. However, there are still imperfections in the diagnostic criteria of PM. The development of muscle imaging led to revisiting not only the PM diagnosis strategy but also the patients’ follow-up.

**Teaching point:** PM should be considered and included in the differential diagnosis of a patient with inflammatory signs and muscular pain, and the radiologist should be aware of its imaging features.

## Introduction:

The symptoms of PM are variable and not specific. These can mimic many other diseases. A high suspicious index and a good correlation of the clinical history combined with the radiological findings help to reach optimal diagnosis conditions and the avoidance of other unnecessary investigations and treatments.

## Case History

A 41-year-old female patient was admitted in the emergency department complaining of diffuse muscle aches, general muscle weakness (especially the upper girdles) transpiration and shivering.

Physical examination showed a pale patient with a painful palpation of the left shoulder and a temperature of 37.8°C. No other abnormality was determined; more specifically no skin lesions were noted. Laboratory examination showed: C-reactive protein (CRP) 147, erythrocyte sedimentation rate (ESR) 28, and WBC 23,800. The levels of creatine kinase (CK) 5712, alanine aminotransferase (ALT), aspartate aminotransferase (AST) and lactate dehydrogenase (LDH) were elevated. Autoimmune markers like antinuclear antibodies (ANA) and anti-OJ (anti-isoleucyl-tRNA synthetase) autoantibodies were positive.

**Figure 1:**
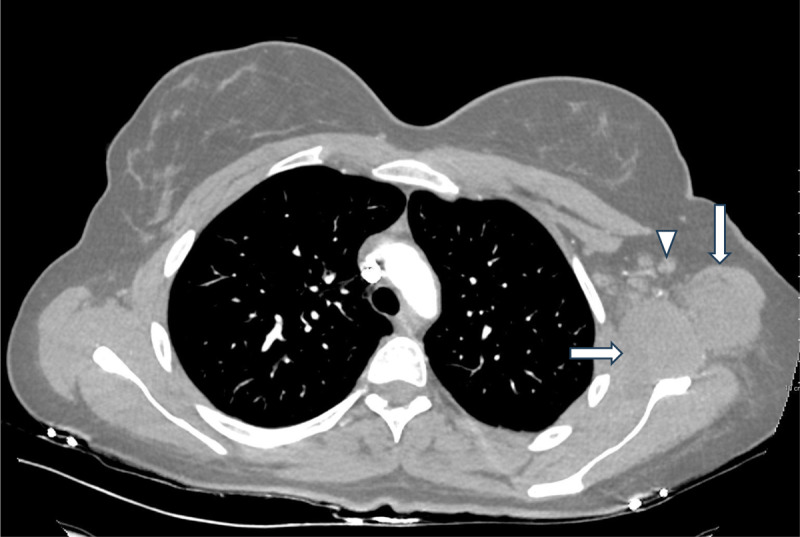
CT of the thorax showing swelling of the muscles of the left shoulder girdle (arrows) with surrounding fat infiltration and small adenopathy (arrow head) in the left axilla.

Computed tomography (CT) scan of the thorax and the abdomen showed a swelling of the muscles of the left shoulder girdle with a surrounding fat infiltration (Figure 1, arrows) and a small adenopathy in the left axilla (Figure 1, arrow head). Inflammatory or infectious PM was suspected according to elevated CKs, clinical and CT findings.

**Figure 2:**
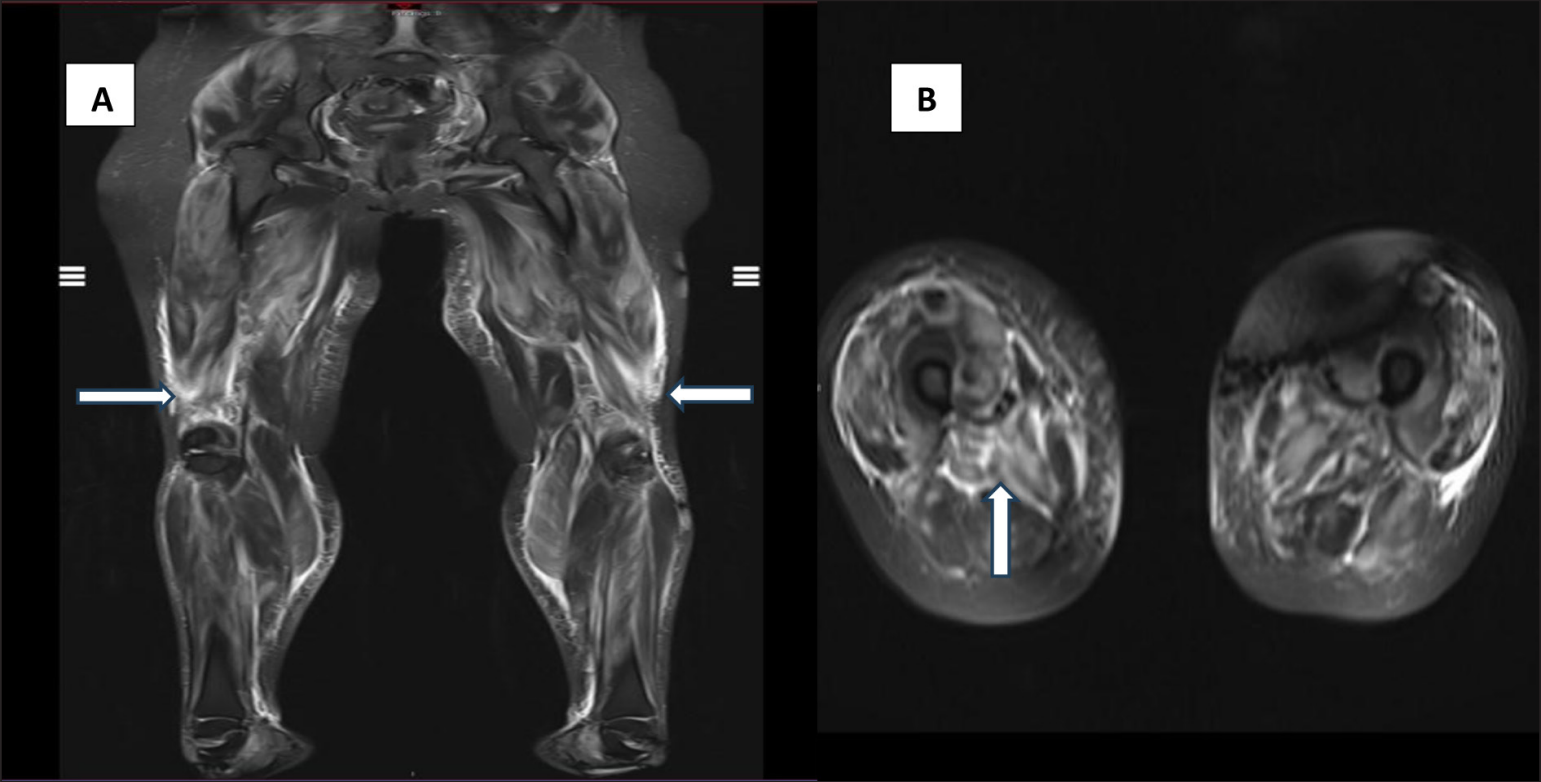
Coronal A and axial B short tau inversion recovery (STIR) images showed diffuse hyper intensity in thigh muscles (arrows). Note that there is no associated increase in signal in the subcutaneous tissue and skin thickening that occur in dermatomyositis.

Magnetic resonance imaging (MRI) of the lower limbs in our patient revealed a diffuse heterogonous muscular edema without involution or atrophy. These results support the diagnosis of myositis (Figure 2 A–B arrows).

**Figure 3:**
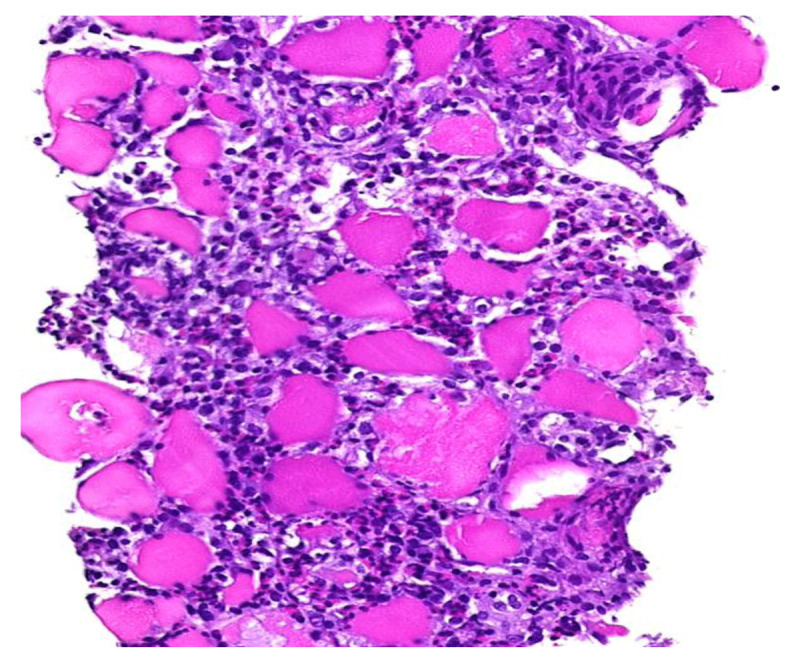
Histopathological examination of muscles of the right upper thigh revealed an unspecified inflammatory muscle pathology (myositis) rich in eosinophils and lymphocytes.

Muscle biopsy of the right upper thigh revealed an inflammatory muscle pathology (myositis) rich in eosinophils and lymphocytes (Figure 3).

**Figure 4:**
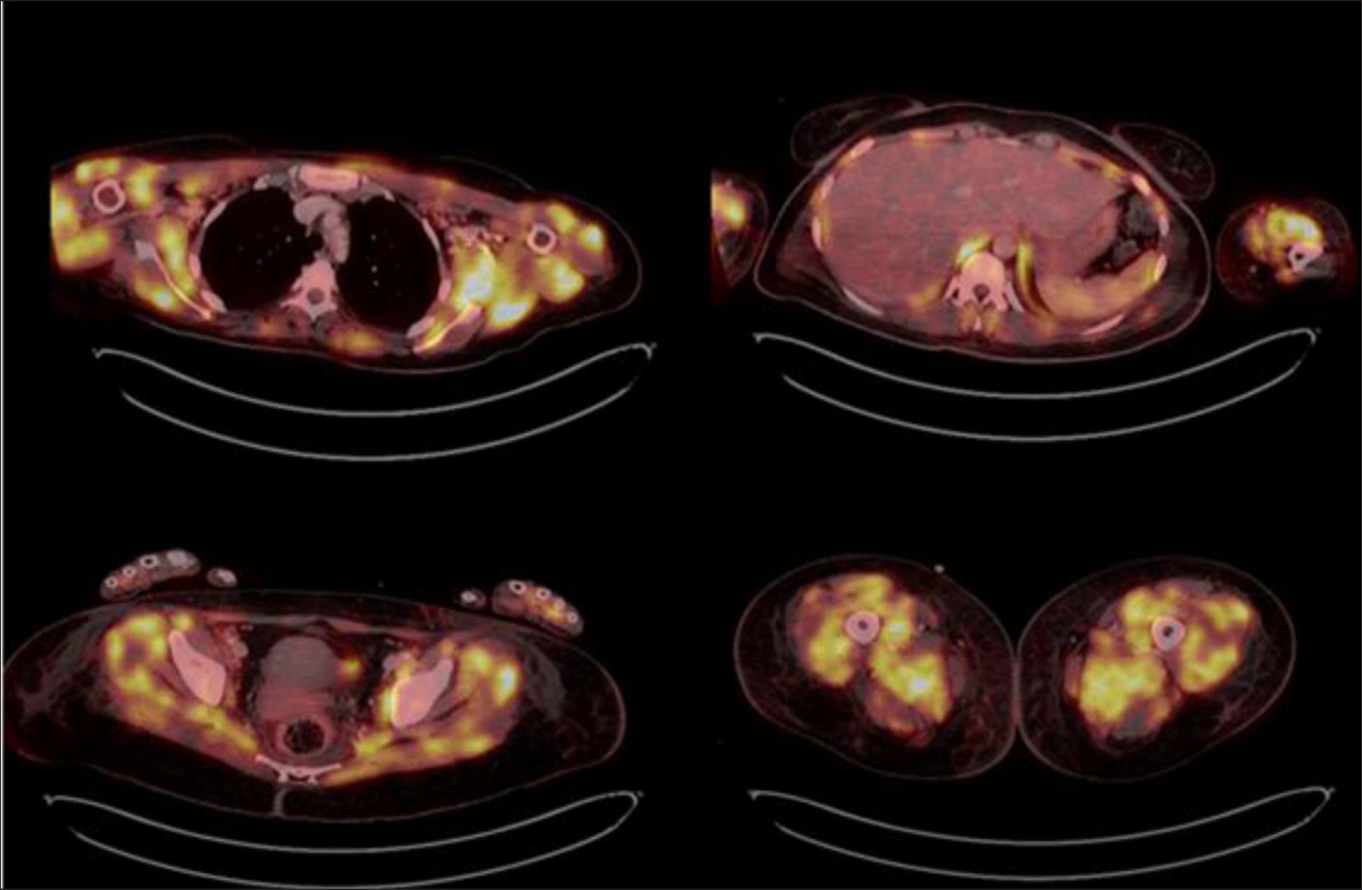
Diffuse FDG uptake in the shoulder, hip girdle muscles, as well as in the intercostal and lower limb muscles.

PET-CT showed increased FDG uptake in all muscle groups, which is consistent with myositis (Figure 4).

Results of the electromyography test (EMG) were compatible with myogenic suffering, possibly consistent with myositis.

## Comments

Clinically, PM manifests itself as symmetrical and progressive proximal muscle weakness of variable intensity. Laboratory test abnormalities are common, especially an increase in CK and LDH. Anti-OJ autoantibodies are rare myositis-specific autoantibodies that have been described to target isoleucyl-tRNA synthetase. In most cases of myopathy, spontaneous activity is increased in EMG test, but it is critical to remember that an entirely normal EMG does not exclude the presence of myopathy.

PM remains a diagnosis of exclusion, and other conditions, such as dermatomyositis, immune-mediated necrotizing myopathy, overlap myositis, and sporadic inclusion body myositis, should be excluded [1].

Multimodal imaging provided new insights into the pathogenesis, diagnosis, therapeutic evaluation, and progression of PM. MRI is the most important imaging modality for the assessment of inflammatory myopathy. The most common pattern of MRI in PM is bilateral and symmetrical muscular edema in the pelvic girdle and thighs with preservation of the muscular architecture [2]. The signal intensity of the edema is directly proportional to the inflammatory status and the severity of the disease [3].

Myositis can be associated in 20% of cases with interstitial lung disease, which may lead to complications such as pulmonary hypertension and may be fatal under rapidly progressive conditions. CT allows the detection of pulmonary changes prior to the appearance of clinical symptoms. High-resolution CT is sensitive to the specific interstitial pneumonitis appearances of ground-glass opacities and honeycombing [4].

The association between malignancy and inflammatory myopathy has been supported by numerous epidemiologic studies; therefore, all patients newly diagnosed with PM should be evaluated for the possibility of an underlying malignancy [5].

Therefore, PET-CT has multiple applications in this disease, starting with cancer screening as well as the ability to measure the activity of the disease in muscle. It can also help to differentiate between myositis phenotypes.

## Conclusion

Diagnostic imaging plays an important and interesting role in the detection, evaluation, and follow-up of inflammatory myositis.
